# A Longitudinal Low Dose *μ*CT Analysis of Bone Healing in Mice: A Pilot Study

**DOI:** 10.1155/2014/791539

**Published:** 2014-11-06

**Authors:** Lu-Zhao Di, Vanessa Couture, Élisabeth Leblanc, Yasaman Alinejad, Jean-François Beaudoin, Roger Lecomte, François Berthod, Nathalie Faucheux, Frédéric Balg, Guillaume Grenier

**Affiliations:** ^1^Research Center of CHUS (CRCHUS), 3001, 12th Avenue North, Sherbrooke, QC, Canada J1H 5N4; ^2^Department of Orthopedic Surgery, Faculty of Medicine and Health Sciences, University of Sherbrooke, 3001, 12th Avenue North, Sherbrooke, QC, Canada J1H 5N4; ^3^Sherbrooke Molecular Imaging Centre (CIMS), CRCHUS, 3001, 12th Avenue North, Sherbrooke, QC, Canada J1H 5N4; ^4^Department of Nuclear Medicine and Radiobiology, Faculty of Medicine and Health Sciences, University of Sherbrooke, 3001, 12th Avenue North, Sherbrooke, QC, Canada J1H 5N4; ^5^Laboratoire d'Organogénèse Expérimentale (LOEX), Research Center of CHUQ, Enfant-Jésus Hospital, 1401, 18th Street, Quebec City, QC, Canada G1J 1Z4; ^6^Department of Surgery, Faculty of Medicine, Laval University, 2325 Université Street, Quebec City, QC, Canada G1V 0A6; ^7^Department of Chemical and Biotechnological Engineering, Faculty of Engineering, University of Sherbrooke, 2500 Université Boulevard, Sherbrooke, QC, Canada J1K 2R1

## Abstract

Low dose microcomputed tomography (*μ*CT) is a recently matured technique that enables the study of longitudinal bone healing and the testing of experimental treatments for bone repair. This imaging technique has been used for studying craniofacial repair in mice but not in an orthopedic context. This is mainly due to the size of the defects (approximately 1.0 mm) in long bone, which heal rapidly and may thus negatively impact the assessment of the effectiveness of experimental treatments. We developed a longitudinal low dose *μ*CT scan analysis method combined with a new image segmentation and extraction software using Hounsfield unit (HU) scores to quantitatively monitor bone healing in small femoral cortical defects in live mice. We were able to reproducibly quantify bone healing longitudinally over time with three observers. We used high speed intramedullary reaming to prolong healing in order to circumvent the rapid healing typical of small defects. Bone healing prolongation combined with *μ*CT imaging to study small bone defects in live mice thus shows potential as a promising tool for future preclinical research on bone healing.

## 1. Introduction

Bone healing is a constantly growing field of research with important clinical implications. Precise and effective monitoring of bone healing is thus essential. Histological preparations are traditionally used to assess tissue composition and bone repair processes. However, they are limited to two-dimensional analyses of three-dimensional (3D) structures and semiquantitative scoring systems. Biomechanical testing is also a reliable quantitative method for assessing bone repair but is limited to endpoint analyses, as are histological preparations [1]. Because of high inter- and intrasubject variability, such studies involving several time points require large numbers of animals, which increases the cost of labor-intensive experiments [2].

The use of *µ*CT in experimental medicine has increased exponentially over the past few years because this imaging technique provides reliable and highly reproducible results [3]. *µ*CT imaging enables both noninvasive, tissue-preserving imaging and quantitative 3D morphometry of bone structures, notably of woven bone [4, 5], and has thus rapidly evolved to become the gold standard for microarchitecture analyses [6, 7]. Longitudinal low dose *µ*CT analyses have been used to study treatments for enhancing the healing of calvarial defects in murine models [8–11] and to quantify tumor-induced osteolysis [12]. However, *µ*CT data collection often requires* ex vivo* specimens, meaning that the animals have to be sacrificed [6]. While a few investigators have reported using low dose *µ*CT to study bone architecture* in vivo* [13–15], none has used longitudinal *µ*CT scans to evaluate the healing of drill hole cortical defects in mice, mainly because spontaneous healing occurs quickly, making it difficult to assess the effect of treatments on bone healing [16]. To slow down spontaneous bone healing, Gao et al. irradiated the limbs of mice to reduce the contribution of endogenous cells to bone healing [17], while effective irradiation impairs nonstem cell therapies, such as growth factors that stimulate the recruitment and differentiation of the endogenous progenitors required for bone repair.

In the present study, we used low dose *µ*CT imaging to perform longitudinal quantitative analyses of bone healing of femoral cortical defects in mice. To assess bone healing, we performed longitudinal *µ*CT scans of live animals using a dedicated small-animal low dose *µ*CT scanner and analyzed the images using custom semiautomated segmentation and coregistration software. Despite creating noncritical size femoral defects, our results enabled us to show that intramedullary reaming is effective in slowing down spontaneous bone healing and significantly increasing the time required for the defect to heal. In summary, the combination of low dose *µ*CT scan imaging of live animals and bone reaming appears to be a valuable tool for evaluating the kinetics of long bone healing following treatments with drugs, biomaterials, and/or cells in mice.

## 2. Materials and Methods

### 2.1. Animals

Skeletally mature 12- to 16-week-old male CD1 (Charles River, Saint-Constant, QC, Canada) mice were used. This study was carried out in strict accordance with the recommendations in the Guide for the Care and Use of Laboratory Animals of the National Institutes of Health. The protocol was approved by the Committee on the Ethics of Animal Experiments of the University of Sherbrooke (Permit Number 141-11B). Prior to surgery, the mice received a narcotic (Buprenorphine; 0.05–0.1 mg/kg) to control pain as well as a preventive dose of antibiotic (penicillin G; 60,000 U).

### 2.2. Surgical Procedure for Creating Femoral Cortical Defects and Reaming the Medullar Cavity

**Figure 1:**
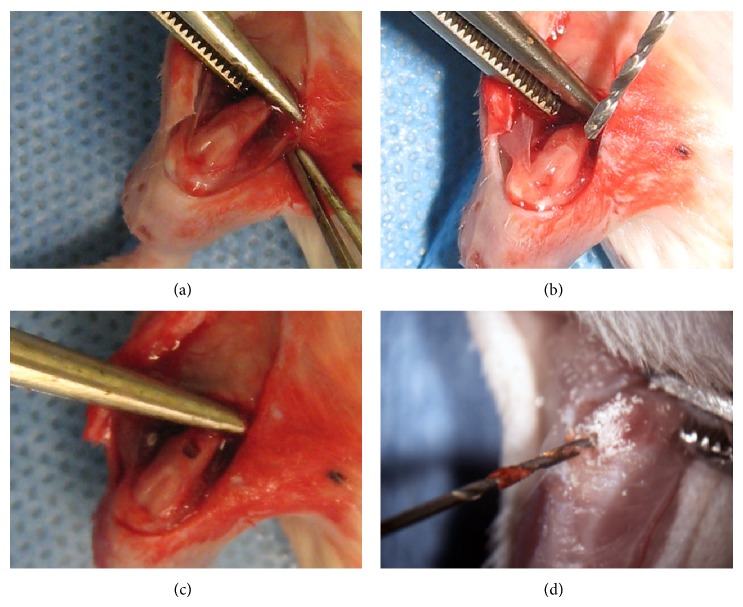
Surgical procedure for creating diaphyseal femoral cortical defects and intramedullary reaming. The femur was exposed (a) and the quadriceps was reclined by everting the patella (b). A cortical defect was created in the distal midshaft portion of the femur using a drill bit (c). Reaming was performed by drilling through the intercondylar notch of the femur (d).

A diaphyseal cortical defect of the anterodistal femur was created using a modified drill hole technique [18, 19] with or without intramedullary reaming as shown in Figure 1. Briefly, the mice were anesthetized (isoflurane), their limbs were shaved, and the skin was disinfected using a 0.5% chlorhexidine solution (Dexidin 0.5; Atlas Laboratory, Montreal, QC, Canada). A skin incision was made to expose the medial quadriceps and knee. A medial parapatellar approach was then made to the knee and was extended proximally through the quadriceps to expose the distal femur by lateral patellar eversion. A 3/64′′ drill bit (Model 865; Dremel, Mount Prospect, IL, USA) was used to create a 1.1 mm-diameter circular defect in the anterior cortex of both femurs 4 mm above the femoral condyles. The defects were irrigated with normal saline to remove bone residues. Intramedullary reaming was performed on one femur by drilling a 1/32′′-hole through the intercondylar notch and then reaming the full length of the medullar cavity. The reamer was kept in the medullar cavity at high speed for 10 s.

The defects were then covered by reclining the quadriceps back to its original position and repositioning the patella. The incision was closed using nonabsorbable sutures. The surgery was performed by an orthopedic surgeon using sterile materials in aseptic conditions. The mice were used for live *µ*CT imaging and/or were euthanized for histological examinations.

### 2.3. Low Dose Microcomputed Tomography (*µ*CT) Scan Imaging

Low dose *µ*CT scan imaging was performed using a Gamma Medica Triumph X-O small-animal CT scanner composed of a 40 W X-ray tube with a 75 *µ*m focal spot diameter and a 2240 × 2368 CsI flat panel X-ray detector. The detector pixel size was 50 *µ*m, and a 2 × 2 pixel binning scheme was used. Scans were performed at 60 kVp and 230 *µ*A using 512 projections in fly mode to reduce exposure with a 59.20 mm FOV. Images were reconstructed using the general purpose reconstruction FBP kernel from the Gamma Medica software over an isotropic 116 *µ*m voxel spacing grid providing a voxel volume of 1.56 × 10^−3^ mm^3^. Air and water phantoms (15 mL Falcon tubes) were scanned for each imaging session to allow normalization in HU. The live mice were scanned once prior to surgery as a control and then at days 0 (immediately after surgery), 7, 14, 21, 30, 35, 44, 57, and 78 after surgery. To minimize movement, the mice were isoflurane-sedated during the *µ*CT imaging. The dose of radiation administered was 11.7 cGy per scan, as measured by dosimeter.

### 2.4. Software to Quantify Bone Healing

Custom software was written to automatically calculate the size of the cortical defects. The *µ*CT scans consisted of 512 individual image slices stacked in a RAW file and converted to the NIFTI format using Fiji freeware (ImageJ, Version 1.42q; National Institutes of Health, USA) [20]. ITK-SNAP freeware (http://www.itksnap.org) was used to segment structures [21] and manually delineate 3D ROI on the cortical defects in the postsurgery (day 0) scans. Tracing an irregular anatomic contour adjacent to the cortical surface was deemed the best approach to create the 3D ROI. We used software provided by the Image Analysis and Visualization Platform (PAVI, Sherbrooke, QC) to virtually extract, align, and save images of each mouse femur to smaller volumes. The virtually extracted femurs were then registered to the day 0 postsurgery reference femur using FSL Flirt [22, 23]. Since we did not want the spatial information of the bones to be lost, we parameterized Flirt to use a rigid body transformation with 6 degrees of freedom. After registration, a linear transformation was applied to the voxel signal intensities to convert them to the HU [24] scale using water for calibration. This allowed the signals from different scans made at different time points to be calibrated. The HU scale makes it possible to conduct analyses of absolute voxel signal intensities in order to determine the material density in each voxel (bone, muscle, water, etc.). We then calculated the kinetics of cortical defect healing using the previously determined 3D ROI, which became relevant for each time point after registration. The volumes and sizes of the cortical defects in treated and control femurs for each mouse were then quantified over time as were their densities based on the HU scale. The HU ranges for empty or soft tissue reaction (e.g., hematoma) (−1000 to +800 HU), woven bone (+1200 to +1900 HU), and compact bone (>2700 HU; comparable to intact bone) were based on published results from other groups [25–27] as well as on results from specific anatomical sites with known tissue compositions.

### 2.5. Statistics

To calculate the sample size required, we used the following repeated sample size estimation formula, (*n* = 2 + *C*(*s*/*d*)^2), where *s* is the standard deviation and *d* is the difference measured, with a *C* of 7.85, a power of 80%, and an alpha of 0.05. The difference *d* was measured between the volumes of the cortical deficits in the left and right femurs on postoperative day 29 when the dissimilarity between the two femurs was the most marked. The standard deviation was also calculated using the results on postoperative day 29. The standard deviation was 0.255, and the measured difference was 0.36. The sample size required for a significant result was thus at least 6 mice.

One outcome of the study was the assessment of the agreement among three observers with respect to their 3D ROI determinations for the same *µ*CT scan. This was represented by a Bland-Altman plot [28]. Graphically, the mean differences and the 95% confidence intervals (CI) were represented. An intraclass coefficient (ICC) was also used to assess their reliability and validity. The ICC for the interobserver agreement was obtained by comparing blind measurements of the three observers. For clinical studies, a Cronbach alpha value over 0.85 is considered very good, while a value over 0.9 is considered “ideal” [29].

The main outcome of the present study was to assess the differences in 3D ROI voxel HU cortical defect volumes between the reamed and unreamed femurs relative to the respective postoperative day 0 volumes. The comparison of means between reamed and unreamed samples at the same time point was analyzed using two-way ANOVA and Sidak's multiple comparisons test. *P* values less than 0.05 were considered significant.

Statistical analyses were performed using SPSS v20.0.0, and the results were graphed using Prism v5.0.

## 3. Results

### 3.1. 3D ROI: Analysis of Interobserver Agreement

**Figure 2:**
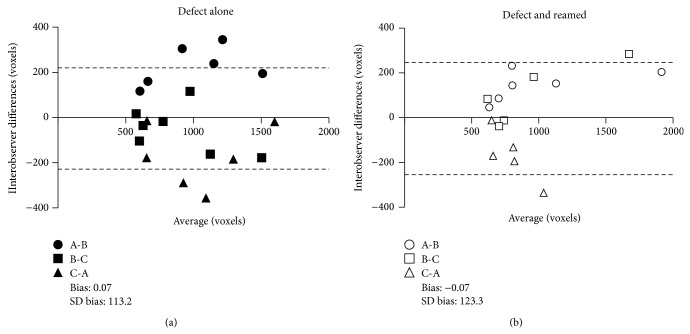
Interobserver agreement in the determination of 3D ROI. A Bland-Altman plot was used to graph differences in the absolute number of voxels per 3D ROI as a function of the average size of the defect. This made it possible to assess the agreement between three observers in the determination of the 3D ROI. Eighteen *µ*CT images from (a) unreamed femurs with a defect and (b) reamed femurs with a defect were blindly analyzed. The two-by-two comparison of the three observers (A-B, B-C, and C-A) showed a 95% confidence interval from −226.5 to +226.4 and from −245.9 to +247.1 for unreamed femurs with a defect and reamed femurs with a defect, respectively. An interclass correlation (ICC) was used to compare interobserver variability. The ICC scores were 0.864 (very good, *P* < 0.0001) and 0.905 (ideal, *P* < 0.0001) for the unreamed femurs with a defect and the reamed femurs with a defect, respectively.

Since initial 3D ROI segmentation may be subjective and thus overestimate or underestimate healing, we verified the interobserver agreement, which was represented using Bland-Altman plots (Figure 2). We used three observers (A-B-C) to blindly determine the 3D ROI of the defects. The results are presented as the difference in the absolute number of voxels per 3D ROI as a function of the average size of the defect. Of the 36 scans, 18 were of femurs with a defect but no reaming (Figure 2(a)) and 18 were of femurs with a defect and reaming (Figure 2(b)). The two-by-two comparison of the three observers (A-B, B-C, and C-A) showed that the 95% confidence interval of the evaluations of the observers ranged from −226.5 to +226.4 (21.9% ± 5.0) and from −245.9 to +247.1 (20.1% ± 3.2) for defects without reaming and defects with reaming, respectively.

To determine whether the interobserver agreement was significant, we calculated an interclass correlation (ICC), which allowed us to compare interobserver variability for a given sample. The ICC was 0.864 (very good, *P* < 0.0001) for the defect without reaming and 0.905 (ideal, *P* < 0.0001) for the defect with reaming, indicating very good interobserver agreement. In addition, there was no significant difference between the 3D ROI of the defect without reaming and the defect with reaming.

### 3.2. Longitudinal Low Dose *µ*CT Analysis of Bone Healing

**Figure 3:**
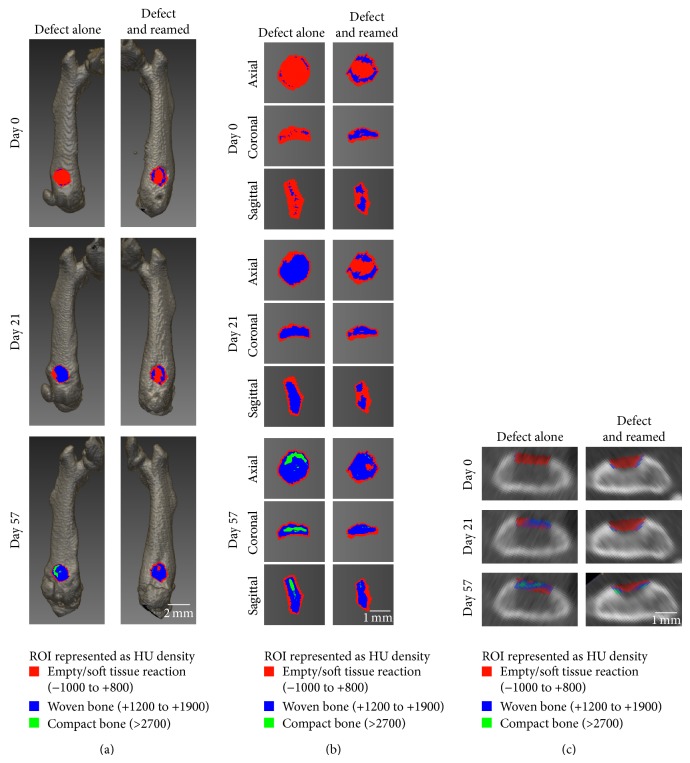
Bone healing monitoring as a function of time using low dose *µ*CT scans of live mice. (a) Representative views of 3D *µ*CT reconstructed images of femurs on days 0, 21, and 57 after surgery. Variations in the density of the 3D ROI (determined at day 0 after surgery) make it possible to estimate the tissue composition. (b) 3D ROI can also be observed in axial, coronal, and sagittal views. (c) Representative cross-sectional views of the femur in which the 3D ROI is apparent in one image (slice). The colored voxels make it possible to see changes in defect composition as a function of time where most of the voxels were red on day 0 and blue on day 28, with an increasing proportion of green on day 57. Nonorganized substrate (red: −1000 to +800 HU), woven bone (blue: +1200 to +1900 HU), and compact bone (green; >2700 HU).

We determined the difference in the kinetics of bone healing between reamed and unreamed femurs by calculating the number of voxels in nonorganized substrate, woven bone, and compact bone.  Figure 3(a) shows reconstructed images of anteroposterior views of the femurs with their defects on days 0 and 21 as well as on day 57 after surgery, when the most significant variations in tissue composition of the reamed and unreamed femurs were observed. In addition to visualizing the registration of the femurs at different times, it is possible to identify the position of the 3D ROIs overlaid on the defects along different views (Figure 3(b)). Cross-sectional views of the femurs can also be seen in which the 3D ROI is apparent (Figure 3(c)). In both representations, the voxels of the 3D ROI are color-coded to differentiate between the various components in the defect, that is, empty/soft tissue reaction (red), woven bone (blue), and compact bone comparable to intact bone (green). Figures 3(b) and 3(c) show that woven bone and compact bone formation is delayed in the reamed preparation compared to the unreamed preparation.

**Figure 4:**
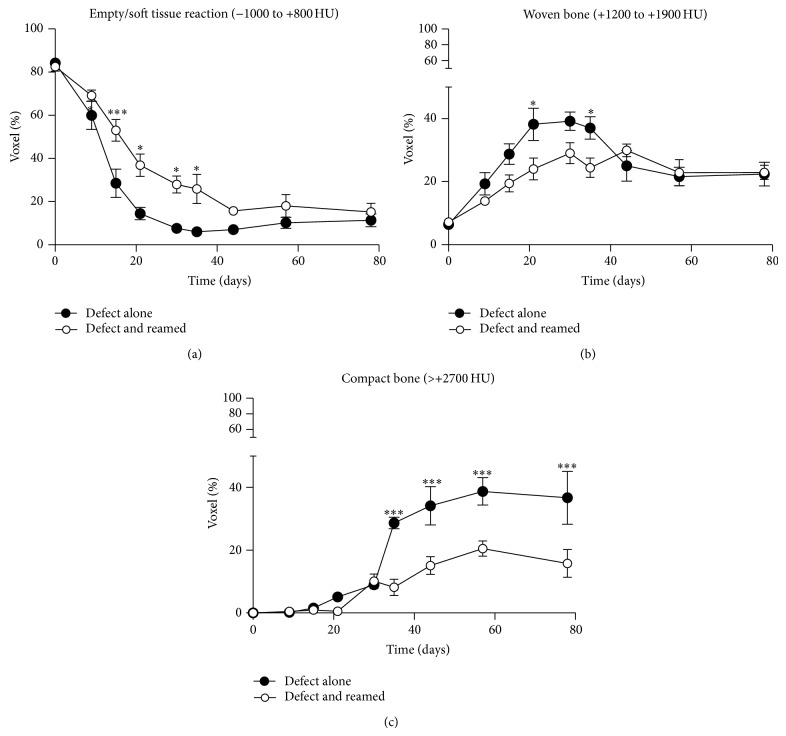
Bone healing as a function of time for a defect without reaming and a defect with reaming. (a) The results were quantified and plotted as a percentage of voxels representing nonorganized material as a function of time for unreamed femurs with a defect and reamed femurs with a defect. The percentages of (b) woven and (c) dense bone formation as a function of time were also graphed. The results were obtained from nine mice (*n* = 9; ^*^ *P* < 0.05, ^***^ *P* < 0.0001).

We used our custom software to verify the filling kinetics of the defects (Figure 4(a)). Graphing the percentage of voxels in the −1000 to +800 HU range within the 3D ROI over time revealed that reaming caused a significant delay in healing. It took 11 and 16 days after surgery for 50% healing to occur in the unreamed and reamed femurs, respectively, while 75% healing took approximately 16 and 36 days, respectively. By day 57, there was no significant difference in healing between the two surgical procedures.

We also assessed the formation of woven bone by graphing the percentage of voxels in the +1200 to +1900 HU range within the 3D ROI as a function of time (Figure 4(b)). The percentage of voxels related to woven bone was significantly higher in the unreamed preparations, with the percentage reaching a plateau around day 29 of 40% and 29% for the unreamed and reamed preparations, respectively. Interestingly, the percentages decreased to 20% by day 57, when no significant difference between the preparations was observed.

We investigated the formation of compact bone by graphing the percentage of voxels in the 3D ROI with >2700 HU as a function of time (Figure 4(c)). The percentage of voxels related to dense bone increased significantly faster in the unreamed femurs than in the reamed femurs. This difference was observed by day 35, when the percentage reached 30% for the unreamed femurs but only 8% for the reamed femurs. The percentage increased until day 57, reaching nearly 40% for the unreamed femurs and nearly 22% for the reamed femurs.

## 4. Discussion

*µ*CT is a precise tool for bone imaging, and in recent decades it has been shown to be the gold standard for studying bone structure [30]. It has been shown to be a relevant tool for craniofacial studies in small rodents, in which it is possible to create critical size bone or nonhealing defects [8–11]. Unfortunately, such critical size defects cannot be created in the long bones of mice without using nails or other materials that can impair *µ*CT imaging [31, 32]. On the other hand, small noncritical size bone defects heal spontaneously and rapidly, which may hinder the effectiveness of experimental treatments [16]. To widen this “therapeutical” window, we reamed the endosteum of the medullar region of the bone. The aim of our study was to evaluate the utility of using longitudinal low dose *µ*CT scans to assess the healing of noncritical size defects (1 mm) in long bones in mice and to investigate the effect of intramedullary reaming on healing times.

Longitudinal studies of small cortical defects using low dose *µ*CT scan are challenging for many reasons. First, bone defects created using small drill bits are subject to operator bias due to differences in the angle at which the drill bit enters the femur and due to micromovements once the bit is fully introduced. This bias must be taken into account given the size of the defects (1 mm), which are much smaller than calvarial defects, which can be up to 5 mm in diameter in mice [8]. Second, small defects are assessed using multiple scans of the animals, which, while immobilized, are not in exactly the same position, which in turn increases the risk of miscalculations. To circumvent these experimental biases, the corresponding femur of each animal was first rigorously aligned in space, making it possible to exactly superpose the 3D ROI. The 3D ROIs at day 0 (after operation) were then set and their coordinates were applied to all time points. The results of each time point were relativized to the initial 3D ROI at day 0 (before and after operation). By applying this procedure, we were able to assess callus formation and mineralization over time by using calibrated voxels expressed in HU that were applied to different tissue composition categories, that is, empty or soft tissue reaction (e.g., hematoma), woven bone, and mineralized compact bone. More importantly, the bone healing process over time described in the present study matched that of previous *µ*CT and histological studies [33–35].

In addition, our results showed that the healing of cortical defects is significantly affected by the endosteal reaming procedure. This was shown by the significant delay in defect filling and the formation of woven and compact bone over time. In terms of compositional analyses, Hayward et al. suggested that the use of contrast-enhanced *µ*CT scans may improve the evaluation of nonmineralized (e.g., cartilage) and mineralized tissues in fracture calluses [36]. From our experience, we had no difficulty in distinguishing between these tissues. This may be due to the ranges of HU that we used to categorize the tissues and the use of the 3D ROI at day 0 (prior to surgery) as a reference. Our software made it possible to follow changes in the tissue density of a healing region as small as 1 mm^3^. Low dose *µ*CT analyses may thus provide a reasonable estimate of the tissue mineralization of healing bone based on HU.

Our study also showed that appropriate analysis software and suitable image resolution limit observer variability. Since our software relies on manual tracings of an initial volume of interest (3D ROI) on native *µ*CT scans, interobserver differences may occur given that the exact delineation of cortical defects depends partly on the judgment of the observers. We hypothesized that the greatest interobserver variability is caused by the semisubjective delineation of the initial cortical defect, which is a bias that may be compounded. However, our ICC interobserver analysis showed that this bias is small for experienced observers and does not affect the results. Alternatives include global and local threshold segmentation, but since our 3D ROI was relatively small and well delineated, we judged that manual tracing segmentation would be the most convenient. This finding also implied that, depending on the type of experiment, investigators should choose the appropriate *µ*CT resolution to maintain an acceptable level of precision.

While low dose *µ*CT scans have limited resolution compared to high dose scans, our study showed that the resolution is sufficient and, importantly, low dose scans avoid the biological risk associated with repeated radiation exposure [37–40]. Laperre et al. reported that they observed no hematological toxicity or radiotoxic effect on bone microarchitecture with triple radiation doses of 43.4 cGy per scan [39], which correspond to a cumulative dose of 130 cGy. In our study, the cumulative dose was under 120 cGy, which is far less than 200 cGy, the dose at which the tumor risk increases in mice, thus making any radiation-induced effect on bone healing unlikely [41].

The creation of defects by drilling a hole in the bone cortex is a widely used and effective model for studying bone healing [16, 35], and it is well known that it leads to spontaneous healing [16, 42]. Many reasons could explain the healing delay observed in the reamed preparations. Previous studies have shown that vascular compromise can induce a significant healing delay or even nonunion in rodent fracture models [43, 44]. Since medullar vascularization accounts for about two-thirds of total bone vascularization [45], it is reasonable to think that its disruption could increase healing time. In addition, a loss of medullar substance and endosteum may also contribute to the delay in healing since they contain progenitor cells that contribute to bone healing [35, 46]. Other groups have used different approaches to slow down spontaneous bone healing. To assess how transplanted MSC contribute to healing cortical bone defects, Gao et al. sublethally irradiated the hindlimbs of mice in order to prevent endogenous MSC from participating in the cortical healing process [17]. Prolonging bone healing by medullar reaming may be a useful and simple procedure for limiting spontaneous healing and widening the window for studying the effect of compounds and exogenous stem cells.

## 5. Conclusion

We showed that low dose *µ*CT scans can be used to quantify and characterize bone healing in live mice. Using 3D ROI coordinates determined immediately following surgery, it was possible to evaluate variations in tissue density within the 3D ROI and show that reaming delays the healing of cortical defects in mice. The proposed experimental approach reduces the number of animals needed, provides quantitative longitudinal results, and is reproducible between different observers. Bone healing prolongation combined with *µ*CT imaging to study small bone defects in live mice thus shows potential as a promising tool for future preclinical research on bone healing.

## Supplementary Material

Figure S1: Bone healing kinetics assessment by three independent observers
Healing kinetics were plotted based on the 3D ROI determinations of three observers. The y-axis is the percentage of cortical defect volume compared to day 0, and the x-axis is time in post-operative days. The solid, dashed, and dotted lines represent the bone healing kinetics based on the 3D ROI determinations of three observers (A, B, and C). Filled and empty symbols represent the defect without reaming and the defect with reaming, respectively. (*n*=9)
