# Supplementary material for: A Longitudinal Low Dose μCT Analysis of Bone Healing in Mice: A Pilot Study
Source: Adv Orthop. 2014 Nov 6;2014:791539. doi: 10.1155/2014/791539 (PMC4241339; doi:10.1155/2014/791539)
Supplement: Supplementary file 1 — Figure S1: Bone healing kinetics assessment by three independent observers Healing kinetics were plotted based on the 3D ROI determinations of three observers. The y-axis is the percentage of cortical defect volume compared to day 0, and the x-axis is time in post-operative days. The solid, dashed, and dotted lines represent the bone healing kinetics based on the 3D ROI determinations of three observers (A, B, and C). Filled and empty symbols represent the defect without reaming and the defect with reaming, respectively. (n=9) [file 791539.f1.pdf]

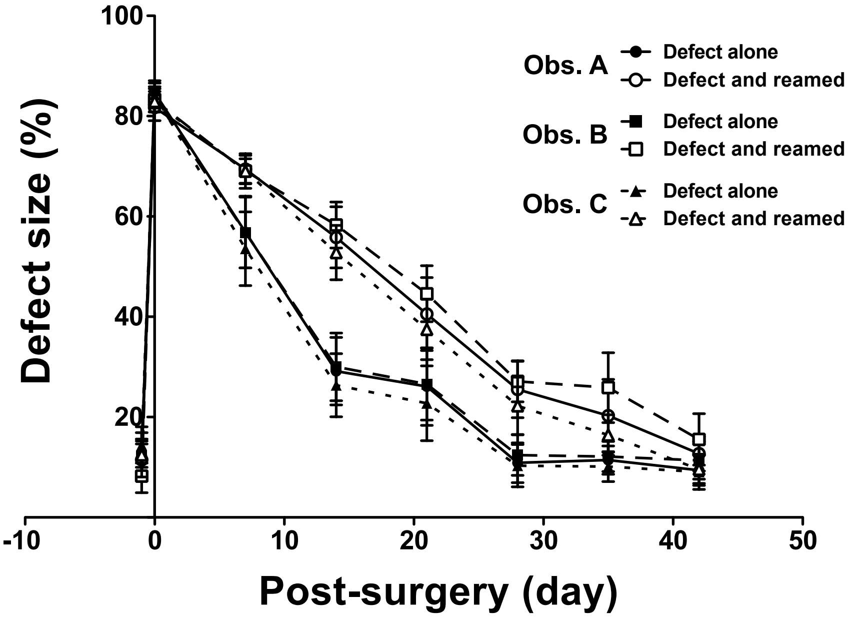

Defect size (%)
100
80
60
40
20
-10
0
10
20
30
40
50
Post-surgery (day)
Obs. A
Defect alone
Defect and reamed
Obs. B
Defect alone
Defect and reamed
Obs. C
Defect alone
Defect and reamed

Figure S1

Figure S1. Bone healing kinetics assessment by three different observers

Healing kinetics were plotted based on the 3D ROI determinations of three observers. The y-axis is the percentage of cortical defect volume compared to day 0, and the x-axis is time in post-operative days. The solid, dashed, and dotted lines represent the bone healing kinetics based on the 3D ROI determinations of three observers (A, B, and C). Filled and empty symbols represent the defect without reaming and the defect with reaming, respectively (n=9). Since the resulting graphs were almost identical, we concluded that there was no significant inter-observer bias.
